# Effect of Early Treatment With Tirofiban on Initial TIMI Grade 3 Flow of Patients With ST Elevation Myocardial Infarction

**DOI:** 10.5812/ircmj.9641

**Published:** 2014-01-05

**Authors:** Mojtaba Salarifar, Mehdi Mousavi, Narges Yousefpour, Ebrahim Nematipour, Seyed Ebrahim Kassaian, Hamidreza Poorhosseini, Alimohammad Hajizeinali, Mohammad Alidoosti, Hassan Aghajani, Younes Nozari, Alireza Amirzadegan, Ali Bozorgi, Yaser Genab

**Affiliations:** 1 Department of Cardiology, Tehran University of Medical Sciences, Tehran Heart Center Hospital, Tehran, IR Iran; 2 Department of Cardiology, Alborz University of Medical Sciences, Shahid Rajai Hospital, Karaj, IR Iran

**Keywords:** Myocardial Infarction, Tirofiban, Percutaneous Coronary Intervention, Angiography

## Abstract

**Background:**

Before primary percutaneous coronary intervention (PCI) in patients with ST elevation myocardial infarction (STEMI), it is not clear whether a routine early administration of glycoprotein IIb/IIIa inhibitors in the emergency ward is beneficial or their administration in selected cases in the catheterization laboratory.

**Objectives:**

The present randomized clinical trial sought to investigate whether an earlier administration of Tirofiban could exert any impact on TIMI grade 3 flows and ST resolution in the electrocardiography of patients with STEMI before primary PCI.

**Materials and Methods:**

Patients with STEMI within twelve hours of symptom commencement were included if primary PCI was planned to be performed within ninety minutes of admission and excluded if they had contraindications for Tirofiban. Seventy patients were randomized to receive 25 μg/kg of bolus Tirofiban early in the emergency ward (the early Tirofiban group) in three minutes and 70 did not receive Tirofiban (the control group). The primary endpoint of the study was a Thrombolysis in Myocardial Infarction (TIMI) grade 3 flows on the initial angiogram. The study is registered as IRCT201105126463N1 in: www.irct.ir.

**Results:**

The study population had a mean age of 57.17 ± 10.09 years and included 79.3 % males. TIMI grade 3 flow was seen in 15 (21.4 %) patients of the Tirofiban group and 7 (10 %) of the control group (P = 0.06, odds ratio = 0.407, and 95 % confidence interval = 0.155-1.072). Complete ST resolution was seen in 30 (42.9 %) patients of the Tirofiban group and 34 (48.6 %) of the control group (P = 0.5).

**Conclusion:**

Although TIMI grade 3 flows trended to be higher in the patients who received early Tirofiban in the emergency ward, the difference did not constitute statistical significance and possible benefits, therefore, require further clarification.

## 1. Background

Percutaneous coronary intervention (PCI) is currently one of the most common and effective treatment modalities for ST elevation myocardial infarction (STEMI) (1). Primary PCI is superior to pharmacological reperfusion therapy on condition that it is immediately available in an experienced center (2). This procedure is recommended in patients with STEMI who can undergo the PCI of the infarct related artery within twelve hours of symptom onset, if performed within ninety minutes of presentation (3).

There are some concerns over the usefulness of a routine use of glycoprotein IIb/IIIa inhibitors in the presence of high-dose clopidogrel (4). Nevertheless, even 600mg clopidogrel may be less effective in patients with STEMI than in those with stable coronary artery disease (5, 6) because during an acute event the absorption of clopidogrel may be impaired (6). Furthermore, pretreatment with acetylsalicylic acid and high-dose clopidogrel alone, might not optimally inhibit platelet aggregation whereas pretreatment with high-dose Tirofiban might be associated with higher platelet aggregation inhibition (7). Glycoprotein IIb-IIIa inhibitors might have such benefits as reducing the likelihood of death in high-risk patients (8) and decreasing ischemic events (3). Thus according to the American College of Cardiology (ACC)/American Heart Association (AHA) guideline, treatment with glycoprotein IIb/IIIa inhibitors is reasonable (class IIa indication) in patients scheduled for primary PCI and treated with unfractionated heparin (UFH) whether or not they are pretreated with clopidogrel (For glycoprotein IIb/IIIa inhibitor administration in patients not pretreated with Clopidogrel, Level of Evidence: A; for glycoprotein IIb/IIIa inhibitor administration in patients pretreated with Clopidogrel, Level of Evidence: C) (3). Although these agents cannot be definitively recommended as routine therapy they might provide more benefit in selective use, such as in patients with large anterior MI and/or large thrombus burden (3).

It seems that various glycoprotein IIb/IIIa antagonists are similarly effective in the setting of primary PCI (3, 9, 10). Abciximab, double-bolus Eptifibatide (180 mcg/kg bolus followed 10 minutes later by a second 180 mcg/kg bolus), and high-bolus dose Tirofiban (25 mcg/kg) all appear to lead to comparable angiographic and clinical outcomes (3). It is not clear whether glycoprotein IIb IIIa inhibitors have any clinical benefit if prescribed to STEMI patient undergoing primary PCI before arrival at the catheterization laboratory (cath-lab) (e.g., ambulance or emergency room) as part of a preparatory pharmacological strategy (1).

Two meta-analyses (11, 12) as well as some other studies (13-16) have shown that an early administration of glycoprotein IIb/IIIa inhibitors confers a higher Thrombolysis In Myocardial Infarction (TIMI) grade 3 flow, while some other studies have not reported significantly higher TIMI grade 3 flow (17-21). ST-segment resolution may also increase with an early administration of glycoprotein IIb/IIIa inhibitors (17, 19). The infarct size, as measured by single photon emission computed tomography, has been reported to decrease with an early administration of Tirofiban (13). Nevertheless, BRAVE 3 trial reported no effect for the early administration of Abciximab (22). 

## 2. Objectives

The present randomized clinical trial sought to investigate whether an earlier administration of Tirofiban could exert any impact on TIMI grade 3 flows and ST resolution in the electrocardiography of patients with STEMI before primary PCI.

## 3. Materials and Methods

Inclusion and exclusion criteria: Patients with a diagnosis of STEMI (ST elevation > 1 mm in two adjacent limb leads or >2 mm in two precordial leads) were included in the study if they presented to the emergency department within twelve hours of symptom onset and if it was anticipated that primary PCI could be performed within ninety minutes after admission. Excluded patients were those with a history of cerebrovascular accident (CVA), transient ischemic attack (TIA) within the previous thirty days or any hemorrhagic CVA at any time, known cerebral pathology (e.g. neoplasm or aneurism), active bleeding or history of bleeding in the previous thirty days, uncontrolled hypertension (systolic blood pressure >180 mm Hg or diastolic blood pressure > 110 mm Hg), major surgery or trauma within the previous six weeks, platelets < 100000/mcl or known bleeding diathesis, coagulation disorder (international normalized ratio [INR] > 1.5), severe hepatic failure, fibrinolytic therapy within twenty-four hours of randomization, or renal failure (creatinine < 2.5 mg/dl or glomerular filtration rate [GFR] < 30 %). Written informed consents were obtained, and the local ethics committee approved the study.

### 3.1. Randomization and Study Protocol

The patients who met the inclusion criteria were randomized via the block randomization method to the early Tirofiban group and the control group. The early Tirofiban group received 25 μg/kg of bolus Tirofiban (Agrastat, Merck & Co., Inc., Whitehouse Station, NJ, USA) diluted in 200 cc normal saline or dextrose water in three minutes in the emergency ward immediately after randomization, whereas the control group did not receive bolus Tirofiban in the emergency ward. All the patients received 325 mg Aspirin and 600 mg Clopidogrel (Plavix, Sanofi-Winthrop Industry, Quetigny, France) and 5000 u UFH in the emergency ward. Additional UFH was given in the cath-lab if activated clotting time (ACT) was less than 200 seconds and the patients continued to receive Aspirin 80-325 mg daily and Clopidogrel 75 mg daily ( for one year) at the physician’s discretion. Other drugs were prescribed according to the present guidelines and the patient’s condition.

Baseline angiography was performed for both groups and primary PCI was done thereafter if feasible. The choice between the bare-metal stent and the drug-eluting stent was dependent on the characteristics of the lesion and decision of the operator. Tirofiban was prescribed at a dose of 0.15 μg/kg/min infusion for twelve to eighteen hours after PCI. The operators could prescribe glicoprotein IIb/IIIa inhibitors (Tirofiban or Eptifibatide) to the control group after the initial angiography if they deemed it necessary. The initial angiograms and those obtained after primary PCI were reviewed by a single blinded investigator and the TIMI flow (23) and TIMI frame count (23) were measured. The primary endpoint of the study was TIMI grade 3 flow on the initial angiogram of the patient before primary PCI.

Electrocardiograms were recorded on admission, before the procedure in the emergency ward, and within one hour after primary PCI (45 to 75 minutes). ST-segment deviation was measured in all the leads at 60 ms after the J point by a single blinded observer. ST-segment resolution was defined as the ratio between one hour post-PCI values and the initial sum of ST-segment deviation. Complete ST-elevation resolution and ST resolution were defined as >70 % decrease in the sum of ST elevation or ST change. If there was a 30 % to 70 % decrease in the sum of ST elevation or ST change, it was defined as partial ST-elevation resolution or ST resolution; and if it was < 30 %, it was assumed not to be significant. Creatinine kinase MB (CKMB) levels were measured at admission time and eight to twelve hours afterward.

The patients were followed up in the hospital and thirty days after PCI (in the outpatient clinic or by telephone) for mortality, myocardial infarction, CVA, need for urgent revascularization, major and minor bleeding, and hematoma. Major bleeding was defined as > 5 g/dl decrease in the hemoglobin level or need for transfusion, and minor bleeding was defined as < 5mg/dl decrease in the hemoglobin level. Hematoma was considered significant if there was > 5cm hematoma at the access site. Major cardiovascular events (MACE) included cardiovascular mortality, myocardial infarction, CVA, and need for urgent revascularization.

Statistical analysis and sample size calculation: Based on the results of the INTAMI Trial (14), we assumed that TIMI grade 3 flow on the first angiography could be 34 % in the early Tirofiban group and 10 % in the control group and that at least 20 % difference in each group would be clinically significant. Consequently, we concluded that 70 patients were needed in each group with a confidence interval of 95 % and power of 80 %. The statistical analyses were conducted using the statistical software SPSS version 16.0 for Windows (SPSS Inc., Chicago, IL). The data are presented as mean ± standard deviation for the numerical variables and raw numbers and percentages ( %) for the categorical variables. The continuous variables were compared using the Student t-test or nonparametric Mann-Whitney U test whenever the data did not appear to have a normal distribution, and the categorical variables were compared using the Pearson chi-square or the Fisher exact test, as required. P values ≤ 0.05 were considered statistically significant. The study is registered as IRCT201105126463N1 in: www.irct.ir.

## 4. Results

The study recruited 140 patients, divided into two groups of 70 patients. The mean age was 57.17 ± 10.09 years and 79.3 % were male. Tirofiban was prescribed 29.78 ± 26.87 minutes before balloon inflation of PCI. At the operator’s discretion, 50 (71.4 %) patients in the early Tirofiban group received maintenance Tirofiban and the others did not. Of the control group, 23 (32.9 %) patients received Tirofiban and 4 (2.86 %) received Eptifibatide in the cath-lab at the operator’s discretion. PCI was not performed in 5 patients after the initial angiography (4 in the early Tirofiban group and one in the control group). The diagnosis of MI was wrong in 2 patients (one in each group).

The baseline characteristics of both groups are depicted in (Table 1), and the clinical, electrocardiographic, and angiographic findings of the two groups are presented in Table 2. TIMI grade 3 flow was detected in 15 (21.4 %) patients in the early Tirofiban group and 7 (10 %) in the control group (Table 2), (P = 0.06, odds ratio = 0.407, and 95 % confidence interval = 0.155-1.072). Multivariate analysis showed that the most important predictors of TIMI grade 3 flows on initial angiography were the involvement of the left circumflex coronary artery (LCX) territory, cigarette smoking, and Tirofiban administration in the emergency department (Table 3). Complete ST-elevation resolution and complete ST resolution were seen in 26 (37.1 %) and 30 (42.9 %) of the early Tirofiban group patients and 29 (41.4 %) and 34 (48.6 %) of the control group patients, respectively (P = 0.604 and P = 0.5).

**Table 1.** Baseline Characteristics of the Tirofiban Group and Control Group ^a^

	Total (n = 140)	Early Tirofiban (n = 70)	Control Group (n = 70)	P Value
**Age, y**	57.2 ± 10.09	55.7 ± 8.62	58.6 ± 11.24	0.09
**Male sex**	111 (79.3)	59 (84.3)	52 (74.3)	0.14
**Current smoker**	62 (44.3)	33 (47.1)	29 (44.1)	0.5
**Diabetes mellitus**	40 (28.6)	18 (25.7)	22 (31.4)	0.45
**Hypertension**	50 (35.7)	23 (32.9)	27 (38.6)	0.48
**Hyperlipidemia**	37 (26.4)	17 (24.3)	20 (28.6)	0.57
**Symptom onset to emergency ward admission time (minute)**	178.1 ± 148.5	185.5 ± 149.8	170.8 ± 147.8	0.4
**Emergency ward admission to balloon time (Door to balloon, minute)**	69.96 ± 62.13	62.2 ± 49.3	77.7 ± 72.3	0.38
**Symptom onset to balloon time**	248.1 ± 161.3	247.7 ± 159	248 ± 164.3	0.99
**Infarct related artery**				
**LAD or diagonal** ^**b**^ ** or diagonal**	60 (42.9)	31 (44.3)	29 (41.4)	0.73
**LCX , OM or Ramus** ^**b**^ **, OM** ^**b**^ ** or Ramus**	24 (17.1)	12 (17.1)	12 (17.1)	1
**RCA** ^**bbb**^ **, PDA or PLV** ^**b**^ ** or PLV** ^**b**^	53 (37.9)	25 (35.7)	28 (40)	0.60
**SVG** ^**b**^ ** graft**	3 (2.1)	2 (2.9)	1 (1.4)	1
**PCI** ^**b**^ ** Characteristics**				
**PCI not performed** ^**b**^ ** not performed**	5 (3.6)	4 (5.7)	1 (1.4)	0.37
**Unsuccessful PCI** ^**b**^	2 (1.4)	2 (2.9)	0	0.5
**Only thrombectomy performed**	2 (1.4)	1 (1.4)	1 (1.4)	1
**POBA** ^**b**^	18 (12.9)	6 (8.6)	12 (17)	0.13
**Stent placement**	113 (80.7)	57 (81.4)	56 (80.1)	0.83
**Bare metal stent placement**	71 (50.7)	30 (42.9)	41 (58.6)	0.063
**Drug eluting stent placement**	42 (30)	27 (38.6)	15 (21.4)	0.027
**Thrombectomy performed**	31 (22.1)	16 (22.9)	15 (21.4)	0.84
**Lesion length (mm)**	25.3 ± 9.1	25.8 ± 9	24.9 ± 9.3	0.54
**Lesion diameter (mm)**	3.13 ± 0.44	3.19 ± 0.44	3.08 ± 0.43	0.29

^a^Data are shown as Numbers ( %) or Mean ± SD.

^b^Abbreviations: LAD: left anterior descending artery, LCX: left circumflex artery, OM: obtus marginalis artery, PDA: posterior descending artery, PCI: percutaneous coronary intervention, PLV: posterior Left ventricular branch, POBA: percutaneous balloon only angioplasty, Ramus: ramus intermedius artery, RCA: right coronary artery, SVG: saphenus vein graft.

**Table 2.** Clinical, Electrocardiographic, and Angiographic Findings in the Early Tirofiban and Control Groups ^a^

Variable	Total (n=140)	Early Tirofiban Group (n = 70)	Control Group (n = 70)	P Value
**TIMI^b^ grade 3 flow in first angiography**	22 (15.7)	15 (21.4)	7 (10)	0.063
**TIMI grade 2 or 3 flow in first angiography**	44 (31.4)	26 (37.1)	18 (25.7)	0.14
**TIMI grade 0 flow in first angiography**	79 (56.4)	28 (40)	33 (47.1)	0.394
**TIMIframe count in first angiogram**	19.98 ± 9.75	20.36 ± 10.44	19.44 ± 8.99	0.76
**TIMI grade 3 flow after procedure**	116 (82.9)	55 (78.6)	61 (87.1)	0.178
**TIMI grade 0 flow after procedure**	7 (5)	4 (5.7)	3 (4.3)	1
**TIMI grade 2 or 3 flow after procedure**	116 (82.9)	55 (78.6)	61 (87.1)	0.18
**TIMI** **frame count after procedure**	12.66 ± 8.34	13.89 ± 9.46	11.45 ± 6.94	0.139
**ST elevation resolution (percent)**	54.76 ± 36.64	56.44 ± 34.43	53.08 ± 38.91	0.317
**ST resolution (percent)**	59.65 ± 33.55	62.5 ± 30.75	56.8 ± 36.13	0.59
**Complete ST elevation resolution (> 70 %)**	55 (39.3)	26 (37.1)	29 (41.4)	-
**Partial ST elevation resolution (30-70 %)**	53 (37.9)	30 (42.9)	23 (32.9)	-
**No significant ST elevation resolution (< 30 %)**	32 (22.9)	14 (20)	18 (25.7)	0.452
**Complete ST resolution (> 70 %)**	64 (45.7)	34 (48.6)	30 (42.9)	-
**Partial ST resolution (30-70 %)**	50 (35.7)	25 (35.7)	25 (35.7)	-
**No significant ST resolution (< 30 %)**	26 (18.6)	11 (15.7)	15 (21.4)	0.649
**Any bleeding complication (Hematoma, Major or minor bleeding)**	13 (9.3)	7 (10)	6 (8.6)	0.771
**Hematoma**	10 (7.1)	6 (8.6)	4 (5.7)	-
**Major bleeding**	2 (1.4)	1 (1.4)	1 (1.4)	-
**Minor bleeding**	1 (0.7)	0	1 (1.4)	-
**30 days MACE** ^**b**^	7 (5)	2 (2.9)	5 (7.1)	0.441
**Cardiac death**	3 (2.1)	0	3 (4.3)	-
**CABG** ^**b**^	4 (2.9)	2 (2.9)	2 (2.9)	-

^a^Data are shown as Numbers ( %) or Mean ± SD.

^b^Abbreviations: CABG: coronary artery bypass graft, MACE: major adverse cardiovascular events, TIMI grade flow: coronary flow according to Thrombolysis In Myocardial Infarction grading system (23).

**Table 3.** Final Step of The Multivariate Analysis using Logistic Regression With the Backward Wald Method for the Predictors of TIMI Grade 3 Flows in the Initial Angiography. Variables in the Model Included Age, Sex, Cigarette Smoking, Involvement of Left Circumflex or Right Coronary Artery Territory, and Early Tirofiban Administration in the Emergency Department

Variable	Ex B	95 % Confidence Interval For Ex B	P value
**Involvement of LCX^a^ territory**	0.300	0.103 – 0.874	0.027
**Cigarette smocking**	0.354	0.130 – 0.964	0.04
**Early tirofiban administration in emergency department**	0.407	0.148 – 1.115	0.06

^a^Abbreviations: LCX: left circumflex artery

Thirty days' follow-up was completed for all the patients. A comparison between the clinical findings at thirty days' follow-up of the patients who received glycoprotein IIb/IIIa inhibitors and those of the patients who did not receive the drug is given in Table 4. 

**Table 4.** Clinical Follow-up and Complications in the Patients who Received Glycoprotein IIb/IIIa Inhibitors in Contrast to Those Who did not Receive the Drug

	Total (n = 140)	Bolus glycoprotein IIb/IIIa inhs in cath-lab^a^ or emergency department (n = 97)	No glycoprotein IIb/IIIa inhs^a^ (n = 43)	P value
**Any bleeding complication (Hematoma, Major or minor bleeding)**	13 (9.3)	9 (9.3)	4 (9.3)	1
**Hematoma**	10 (7.1)	6 (6.2)	4 (9.3)	0.72
**Major bleeding**	2 (1.4)	2 (2.1)	0	-
**Minor bleeding**	1 (0.7)	1 (1)	0	-
**30 days MACE** ^**a**^	7 (5)	4 (4.1)	3 (7)	0.676
**Cardiac death within 30 days**	3 (2.1)	1 (1)	2 (4.7)	-
**CABG** ^**a**^ ** within 30 days**	4 (2.9)	3 (3.1)	1 (2.3)	-

^a^Abbreviations: CABG: coronary artery bypass graft, Cath-lab: catheterization laboratory, Glycoprotein IIb/IIIa inh: glycoprotein IIb IIIa inhibitors, MACE: major adverse cardiovascular event

## 5. Discussion

Complete restoration of anterograde perfusion by TIMI grade 3 flow (23) in patients with STEMI undergoing reperfusion therapy is associated with the lowest mortality rate (23, 24). TIMI grade 3 flow is far superior to grade 2 in terms of short- and long-term survival benefit and reduction in the infarct size (2). Patients from ADMIRAL (15) and PAMI (25) Trials who had TIMI grade 3 flow before primary PCI had improved early and late survival and were less likely to develop complications related to left ventricular failure (25). Primary endpoint of our study was, therefore, to compare TIMI grade 3 flow on the initial angiography of STEMI patients as an indicator of vessel patency before primary PCI.

TIMI grade 3 flow on initial angiography was higher in our study patients who received high dose bolus Tirofiban in the emergency ward, but this finding did not reach statistical significance (15 patients (21.4 %) in the early Tirofiban group and 7 (10 %) in the control group, Table 2), (p value =0.06, odds ratio = 0.407, and 95 % confidence interval = 0.155-1.072).

A meta-analysis (six trials, N = 931) of patients undergoing primary PCI receiving Abciximab (three trials) or Tirofiban (three trials) reported that an early glycoprotein IIb/IIIa inhibitor administration at initial contact (emergency department or ambulance), compared with that in the cath-lab, resulted in a higher TIMI-3 flow rate (20.3 % [84/413] vs. 12.2 % [51/418]) (12). Another meta-analysis used data from 1662 patients randomly assigned to early and late glycoprotein IIb/IIIa inhibitors and showed that an early glycoprotein IIb/IIIa inhibitor administration was associated with a higher pre-procedural TIMI grade 3 flow (11). Post-procedural TIMI grade 3 flow and myocardial blush grade 3 also trended to be higher with early glycoprotein IIb/IIIa inhibitors but did not reach statistical significance except for Abciximab (11). There are other studies that have shown that an early administration of glycoprotein IIb/IIIa inhibitors (Tirofiban (13, 16), Eptifibatide in INTAMI Trial (14) and Abciximab in ADMIRAL trial (15) might improve initial coronary flow.

The ON-TIME Trial randomized primary PCI patients to early, pre-hospital initiation of low dose Tirofiban (10 mcg/kg followed by a maintenance infusion 0.15 mcg/kg/min) (early), and initiation in the cath-lab and found that TIMI 3 flow was present more, albeit not significantly, in the early group on initial angiography (19 % in the early group and 15 % in the late group, p value = 0.22). There was, however, no difference with respect to TIMI grade 3 flow or the myocardial blush grade between the groups, after PCI (18). In some other studies, the improvements have been statistically non-significant (17, 19-21). More clinical trials are needed to make sound judgment on the effects of an early administration of Tirofiban or other glycoprotein IIb/IIIa inhibitors on the initial coronary flow before primary PCI.

Some studies (12, 26), in contrast to others (20), have suggested that an early administration of glycoprotein IIb/IIIA inhibitors in the emergency department (12, 26) or ambulance (20) before primary PCI is associated with higher TIMI 2 or 3 flow in conjugation with higher TIMI myocardial perfusion (21) and lower (faster) initial TIMI frame count (21) than its administration in the cath-lab. In our study, TIMI grade 2 or 3 flow on initial angiography trended to be higher in the early Tirofiban group, but this was not statistically significant (Table 2). Initial TIMI frame count also was not statistically different between our two groups.

Electrocardiographic ST-segment resolution can evaluate the adequacy of myocardial perfusion and strongly predict the outcome in STEMI (2). Even in the presence of TIMI grade 3 flow after primary PCI, absence of ST resolution can reflect inadequate myocardial perfusion, and it identifies patients with a higher risk of left ventricular dysfunction and mortality, presumably because of micro vascular damage in the infarct zone (2). Earlier reperfusion (27) and thrombosuction (28) of an intra-coronary thrombus has a clear relationship with ST resolution and mortality. In the ONTIME 2 Trial, patients who received high-dose Tirofiban at first medical contact (25 mcg/kg bolus followed by 0.15 mcg/kg per min for eighteen hours) benefited from improved ST-segment resolution before and one hour after PCI compared with controls, who received placebo at first and bail-out Tirofiban at the cath-lab. Also, TIMI grade 0 flow was significantly less in the early Tirofiban group (19). In this study, early stent thrombosis was also significantly decreased at thirty days (29). There are other studies suggesting that an early administration of Tirofiban might be associated with higher ST resolution (11, 17), while there are some other studies that have not reached such a conclusion (14, 20).

In our study, complete ST resolution was seen in 30 (42.9 %) of the early Tirofiban group and 34 (48.6 %) of the control group (p value = 0.5), and it seems that an early administration of Tirofiban exerted no significant impact on ST resolution. This might be because 35.76 % of our control group received bail-out glycoprotein IIb/IIIa inhibitors in the cath-lab at the operator’s discretion. Moreover, our study is not sufficiently powered to discuss the effectiveness of glycoprotein IIb/IIIa inhibitors with regard to ST resolution. Thirty days’ follow-up in our study demonstrated that MACE occurred in 2 (2.9 %) patients in the early Tirofiban group and 5 (7.1 %) in the control group.

Some studies have shown improvements in the rate of thirty days’ MACE with an administration of Tirofiban (13, 16, 30) or Eptifibatide (26) before primary PCI. Admiral Trial showed an improvement in a composite of death, reinfarction, or urgent revascularization in patients who received early Abciximab (15). In one meta-analysis, mortality was also not significantly different between the groups, except for Abciximab (11). Some other studies have also reported a downward trend in mortality (12, 30). In the ON-TIME 2 Trial, the patients who received high-dose bolus Tirofiban (25 mcg/kg) before primary PCI exhibited a strong trend toward a decrease in mortality (2.2 % vs. 4.1 %, P = 0.051), which was maintained during one year's follow-up (3.7 % vs. 5.8 %, p value = 0.08) (30). In contrast, Abciximab-facilitated PCI in FINESSE Trial improved neither a composite of death from all causes, ventricular fibrillation occurring more than forty-eight hours after randomization, cardiogenic shock, and congestive heart failure during the first ninety days after randomization (31) nor one year's mortality (32). BRAVE 3 Trial reported no effect for the early administration of Abciximab on a composite of death, recurrent myocardial infarction, stroke, or revascularization of the infarct-related artery at thirty days (22) and one year's follow-up (33). ASSIST Trial (4) (early administration of Eptifibatide), HORIZONS-AMI Trial (Abciximab or Eptifibatide plus heparin in contrast to Bivalirudin) (34), and ON-TIME Trial (low-dose bolus Tirofiban (18)) did not also show such clinical benefits.

It seems that an earlier administration of glycoprotein IIb/IIIa inhibitors after symptom onset to STEMI patients augments their efficacy (19-21). In studies which have reported less or no benefit (AGIR-2 (20), BRAVE-3 (22), FINESSE Trial (31), and HARIZON-AMI (34)), the median time of symptom onset to glycoprotein IIb/IIIa administration is relatively late (e.g. >200 minutes). Our patients also seem to have been relatively late in terms of calling for medical advice (mean symptom onset to emergency ward admission time was 178.1 ± 148.5 minutes, Table 1); this may explain the absence of significant ST-segment resolution or benefit with respect to MACE in our study. Nevertheless, documentation of this effect of an early administration of glycoprotein IIb/IIIa inhibitors on mortality and MACE requires further investigation.

In our study, there were 6 (6.2 %) cases of hematoma in the early Tirofiban group and 4 (9.3 %) cases in the control group. Major bleeding was seen in 2 (1.4 %) patients and minor bleeding in one (1 %) patient of the early Tirofiban group. Even after we compared bleeding complications between the patients who received either early Tirofiban or bail-out cath-lab glycoprotein IIb/IIIa inhibitors and those who did not receive glycoprotein IIb/IIIa inhibitors (Table 4), we did not find a significant difference. There are concerns about increased risk of bleeding when glycoprotein IIb/IIIa inhibitors are prescribed early before the arrival of the patient at the cath-lab (3, 4). Increased incidence of hematoma or minor bleeding after early treatment with Abciximab in ADMIRAL Trial was explained by high doses of heparin as well as the use of a sheath which is left in situ for twenty-four hours (24). Be that as it may, in some studies a pre-hospital use of high-dose Tirofiban has been shown to be safe and associated with a low risk of bleeding (12, 19, 21, 35). It is deserving of note, however, that our sample size was not large eanough to consider bleeding risk.

### 5.1. Study Limitations

According to 2011 ACC/AHA guidelines for PCI, a routine administration of glycoprotein IIb/IIIa inhibitors before the arrival of the patient at the cath-lab (e.g., ambulance or emergency department) as part of an upstream strategy for patients with STEMI undergoing primary PCI is a class III recommendation (no benefit , level of evidence: B) (3). The rational for such a recommendation is mostly because a clinical benefit has yet to be established. While some studies have voiced concern over the increased risk of bleeding in the presence of triple antiplatelet therapy, others have not arrived at such conclusions (12, 19, 35). A very careful heparin-dose protocol (e.g., 5000 u in the emergency ward and 2500 u in the cath-lab if ACT is < 200 ms (19)) with routine measurements of ACT at the cath-lab may decrease bleeding risk. Our study was commenced before the publication of this recommendation and at the time of our study design and recruitment, the 2009 guideline recommended that glycoprotein IIb/IIIa inhibitors be regarded as a preparatory pharmacological strategy before the arrival of patients with STEMI at the cardiac cath-lab for angiography and PCI be considered a class IIb (level of evidence: B) recommendation (1).

High-dose bolus Tirofiban of 25 mcg/kg is associated with a higher level of platelet inhibition compared to the previously used 10 mcg/kg bolus dose (3, 19, 36) and has been shown to be as effective as Abciximab (37, 38) and Eptifibatide (36, 39) before PCI. On the other hand, studies on GP IIb/IIIa inhibitors administered as bolus-only during PCI suggest that this strategy may be similar in efficacy and more cost-effective with fewer bleeding complications compared to the conventional bolus-plus infusion (40). We, accordingly, administered a bolus of 25 mcg/kg in the emergency department and performed continuous infusion optionally at the operator’s discretion, so that 71.4 % of our early Tirofiban group received infusion. Some patients might not need infusion because of incorrect diagnoses, unsuccessful PCI, or the operator’s preferences.

Although non significant statistically, the mean door to balloon time seemed to be somehow less more in our tirofiban group (62.2 ± 49.3 min in Tirofiban group versus 77.7 ± 72.3 min in control group P = 0.38). It may be supposed that this might have an influence on our results, however the time from symptom onset and balloon time was almost the same in both groups (247.7 ± 159 min in Tirofiban group versus 248 ± 164.3 min in control group P = 0.99).

Abciximab may be reasonable to administer intra-coronary in patients undergoing primary PCI (ACC/AHA recommendation class IIb, level of evidence B) (3). Intra-coronary Tirofiban (41, 42) and Eptifibatide (42) have also been safely and effectively prescribed via intra-coronary routes in primary PCI. Some of the operators in our study preferred to administer bail-out Tirofiban or Eptifibatide intra-coronary. The small sample size of our study population precludes a discussion on the effectiveness and results of this type of drug administration.

We opted to allow the operators to prescribe bail-out glycoprotein IIb/IIIa inhibitors to the control group in the cath-lab because ethically we could not enforce the operators to deprive their patients of glycoprotein IIb/IIIa inhibitors (3). Needless to say, these drugs might have benefits in some selected cases (e.g. patients with a large thrombus burden or large anterior infarction or those who have not received adequate Thienopyridine loading).
